# Interface Properties of MoS_2_ van der Waals Heterojunctions with GaN

**DOI:** 10.3390/nano14020133

**Published:** 2024-01-05

**Authors:** Salvatore Ethan Panasci, Ioannis Deretzis, Emanuela Schilirò, Antonino La Magna, Fabrizio Roccaforte, Antal Koos, Miklos Nemeth, Béla Pécz, Marco Cannas, Simonpietro Agnello, Filippo Giannazzo

**Affiliations:** 1 National Research Council-Institute for Microelectronics and Microsystems (CNR-IMM), Z.I. Strada VIII 5, 95121 Catania, Italy; ioannis.deretzis@imm.cnr.it (I.D.); emanuela.schiliro@imm.cnr.it (E.S.); antonino.lamagna@imm.cnr.it (A.L.M.); fabrizio.roccaforte@imm.cnr.it (F.R.); simonpietro.agnello@unipa.it (S.A.); filippo.giannazzo@imm.cnr.it (F.G.); 2 HUN-REN Centre for Energy Research, Institute of Technical Physics and Materials Science, Konkoly-Thege ut 29-33, 1121 Budapest, Hungary; koos.antal@ek.hun-ren.hu (A.K.); nemeth.miklos@ek.hun-ren.hu (M.N.); 3 Department of Physics and Chemistry Emilio Segrè, University of Palermo, Via Archirafi 36, 90123 Palermo, Italy; marco.cannas@unipa.it; 4 ATEN Center, University of Palermo, Viale delle Scienze Ed. 18, 90128 Palermo, Italy

**Keywords:** MoS_2_, GaN, interface, DFT, vdW heterostructures, wide-band gap

## Abstract

The combination of the unique physical properties of molybdenum disulfide (MoS_2_) with those of gallium nitride (GaN) and related group-III nitride semiconductors have recently attracted increasing scientific interest for the realization of innovative electronic and optoelectronic devices. A deep understanding of MoS_2_/GaN interface properties represents the key to properly tailor the electronic and optical behavior of devices based on this heterostructure. In this study, monolayer (1L) MoS_2_ was grown on GaN-on-sapphire substrates by chemical vapor deposition (CVD) at 700 °C. The structural, chemical, vibrational, and light emission properties of the MoS_2_/GaN heterostructure were investigated in detail by the combination of microscopic/spectroscopic techniques and ab initio calculations. XPS analyses on as-grown samples showed the formation of stoichiometric MoS_2_. According to micro-Raman spectroscopy, monolayer MoS_2_ domains on GaN exhibit an average *n*-type doping of (0.11 ± 0.12) × 10^13^ cm^−2^ and a small tensile strain (ε ≈ 0.25%), whereas an intense light emission at 1.87 eV was revealed by PL analyses. Furthermore, a gap at the interface was shown by cross-sectional TEM analysis, confirming the van der Waals (vdW) bond between MoS_2_ and GaN. Finally, density functional theory (DFT) calculations of the heterostructure were carried out, considering three different configurations of the interface, i.e., (i) an ideal Ga-terminated GaN surface, (ii) the passivation of Ga surface by a monolayer of oxygen (O), and (iii) the presence of an ultrathin Ga_2_O_3_ layer. This latter model predicts the formation of a vdW interface and a strong *n*-type doping of MoS_2_, in closer agreement with the experimental observations.

## 1. Introduction

In the last decade, molybdenum disulfide (2H-MoS_2_) emerged as the most investigated two-dimensional (2D) semiconductor material of the transition metal dichalcogenides (TMDs) family, due to its unique physical properties, combined to a good chemical stability and its abundance in nature [1]. MoS_2_ crystals (and in general all TMDs) are characterized by strong in-plane bonds between the chalcogen (X) and the transition metal (M) atoms and weak van der Waals (vdW) interactions between the stacked layers [2,3,4,5]. In particular, the 2H-MoS_2_ polytype exhibits a tunable bandgap as a function of the thickness, i.e., an indirect bandgap of 1.2 eV in the bulk form and a direct bandgap of 1.8–1.9 eV for a monolayer (1L) MoS_2_ [6,7]. One layer and few-layers MoS_2_ have been employed as channel materials in field-effect transistors (FET), showing very promising performances in terms of the on/off state current ratio (≥10^8^) and decent mobility values (up to ~200 cm^2^/Vs under proper conditions) [1]. These properties make MoS_2_ one of the potential replacements of Si for the continuation of the Moore scaling law in digital electronics [8]. Furthermore, 2H-MoS_2_ is very appealing for a wide range of More-than-Moore applications [1,9,10,11,12], including sensing [13,14], photocatalysis [15,16], photovoltaics [17,18], and photonics [19,20] until reaching the more exotic spin-valley physics [21,22,23]. 

In this context, the dangling bonds-free MoS_2_ surface allows the creation of several vdW heterostructures by the combination of various 2D materials (2D/2D vdW heterostructures) [24,25,26,27], by integration of MoS_2_ with semiconductor nanowires (1D core-shell heterostructures) and with bulk semiconductors (2D/3D vdW heterostructures) [1,28,29,30,31,32]. In particular, increasing research efforts have been directed in the last years to the integration of MoS_2_ with wide-bandgap (WBG) semiconductors, including silicon carbide (SiC), gallium nitride (GaN), and related group-III nitrides (AlN and AlGaN alloys). The combination of the unique physical properties of MoS_2_ with the robust properties of highly mature WBG semiconductors (such as high breakdown field and electron saturation velocity [33,34]) set the basis for the realization of new heterojunction diodes that exploit the vertical current at the MoS_2_/WBG interface [35,36,37] and of advanced photodetectors covering both the UV and the visible spectral range [38,39,40]. 

2H-MoS_2_ exhibits a very low lattice constant mismatch with the basal planes of 4H-SiC (~2.9%) [41] and 2H-GaN (<1%) [42] crystals, which represents a favorable condition for highly oriented epitaxial growth of MoS_2_ on these hexagonal substrates [43]. Furthermore, the small difference between the thermal coefficient expansion of the MoS_2_/GaN heterostructure (α_MoS2_ − α_GaN_ ≈ 0.97 × 10^−6^ K^−1^ [40,44,45]) permits the reduction of the residual strain induced by the cooling of the system from the higher growth conditions to the room temperature [40]. The promising performances of devices obtained by the integration of MoS_2_ on GaN have been demonstrated by several research groups [46,47]. As an example, innovative heteroepitaxial devices have been recently reported, such as vertical heterojunction devices [48], Esaki tunnel diodes obtained by the combination of degenerate p^+^-MoS_2_ on n^+^-GaN/Si [49], self-powered broadband (UV–vis–NIR) photodetectors [50,51,52,53,54], light emitting diodes [55], and photovoltaics applications [56]. 

Nevertheless, understanding and controlling the interface properties of the MoS_2_/GaN heterostructures represent the key steps to optimize the performances of demonstrated devices and, eventually, to demonstrate new ones. In fact, the interface of such vdW systems plays a crucial role in terms of electronic transport and carrier transfer. As an example, Poudel et al. reported an increase in photoluminescence (PL) emission from MoS_2_ and a consequent decrease from GaN, which was attributed to electron–phonon coupling and energy transfer at the MoS_2_/GaN interface [57]. Furthermore, angle-resolved photoemission spectroscopy (ARPES) measurements performed on *n*-MoS_2_ flakes transferred on *p*-doped GaN displayed a modification of the band structure caused by the formation of an interface dipole of 0.2 eV [58]. Recently, Zhang et at. [59] performed a nitridation of the GaN surface by N_2_ plasma treatment before transferring MoS_2_ on top of GaN. A modification of the MoS_2_/GaN band structure with respect to a not-nitridated surface and a corresponding enhancement of photo-catalytic properties of the heterojunction were demonstrated, which can be exploited for hydrogen generation.

Besides experimental studies, several theoretical investigations based on the density functional theory (DFT) approach have been performed during the last few years to predict the interfacial properties and energy band-alignment in MoS_2_/GaN heterostructures. Most of these simulation studies considered an ideal lattice-matched interface between monolayer MoS_2_ and the Ga-terminated GaN(0001) surface, which resulted in the prediction of a covalent-like bond at the interface [57,59]. These theoretical results contradict the experimental evidence of a van der Waals (vdW) bond between MoS_2_ and GaN, reported by different authors [36,37,42]. Clearly, studies combining experimental investigations and more refined modeling of the interface are necessary to better understand the properties of this heterostructure.

In this paper, we combined experimental characterizations and DFT calculations to provide a detailed evaluation of the MoS_2_/GaN interface structure and the strain, doping, and the optical emission properties of MoS_2_ domains grown by CVD on GaN. X-ray photoelectron spectroscopy (XPS) displayed the formation of stochiometric MoS_2_ ([S]/[Mo] ≈ 2) without the presence of Mo-oxide (MoO_x_) components. Raman mapping showed that the MoS_2_ domains mainly consisted of monolayers, with a small bilayer fraction, consistently with the intense light emission peak revealed by micro-photoluminescence (μ-PL) mapping. Furthermore, an average *n*-type doping of (0.1–0.2) × 10^13^ cm^−2^ and a very low tensile strain of ~0.25% was evaluated by the correlative plot of the E’ and A’_1_ Raman peaks. The obtained strain was in perfect agreement with the one derived by the exciton peak positions obtained by μ-PL spectra. Cross-sectional scanning transmission electron microscopy (STEM) measurements confirmed both the monolayer MoS_2_ thickness, the presence of a van der Waals (vdW) gap at the interface with GaN, and a modification of the topmost GaN layers with respect to the bulk crystal. Finally, we employed DFT calculations to better understand the structural and electronic properties of the interface between 1L of MoS_2_ and GaN. In particular, three configurations of the GaN surface were considered within this heterostructure: (i) an ideal Ga-terminated GaN(0001) surface, (ii) the passivation of Ga terminations with a monolayer coverage of oxygen (O) atoms, and (iii) the presence of an ultrathin Ga_2_O_3_ film on the GaN surface. The first two configurations resulted in a strong covalent bond at the interface, very different from the experimentally observed vdW interaction. On the other hand, the formation of a vdW gap of 3.05 Å and a significant *n*-type doping of 1L of MoS_2_ was predicted in the presence of an ultrathin Ga_2_O_3_ film at the MoS_2_/GaN interface, which is in close agreement with the experimental results.

## 2. Materials and Methods

The starting material for these experiments was an unintentionally doped GaN(0001) template grown on a c-sapphire substrate. The pristine GaN surface showed a low root mean square (RMS) roughness of ~0.3 nm, evaluated from the AFM image in Figure S1 of the Supplementary Materials.

MoS_2_ was grown on a GaN/c-sapphire substrate by single step CVD at a temperature of 700 °C for 10 min at atmospheric pressure. The process was carried out in a quartz tube furnace with two-heating zones, the first employed for the evaporation of the sulfur powders (7–10 mg) at 150 °C, and the second for the evaporation of the MoO_3_ powders (2–3 mg) at 700 °C. The GaN substrate was placed in the second zone of the furnace above the MoO_3_ crucible. The reaction between the S vapors (transported by an Ar flux of 100 sccm) and MoO_3_ vapors occurred in the gas phase close to the GaN surface, resulting in the nucleation and growth of MoS_2_ domains. 

The MoS_2_ domain coverage on GaN was evaluated by scanning electron microscopy (SEM) using a ThermoFisher Scios 2 dual-beam microscope. X-ray photoelectron spectroscopy (XPS) analysis was carried out by using Escalab Xi+ equipment by Thermo Fisher (Waltham, MA, USA), with a monochromatic Al K X-ray source (energy = 1486.6 eV). The spectra were collected at a take-off angle of 90° relative to the sample surface and pass energy of 20 eV. The instrument resolution was 0.45 eV (FWHM of the Ag 3d5/2 peak). The spectra were aligned using C1s (285 eV) as a reference. 

High-resolution transmission electron microscopy (HR-TEM) and high-angle annular dark field scanning transmission microscopy (HAADF-STEM) were carried out with an aberration-corrected Titans Themis 200 microscope by Thermo Fisher. For the cross-sectional analysis, a focused ion beam (FIB) was used to prepare lamellas from the sample. 

Micro-Raman (μ-Raman) spectra were acquired employing both WiTec Alpha equipment by WiTec (Ulhm, Germany) and a Horiba Raman system with a confocal microscope (100×) and with a laser excitation wavelength of 532 nm. The second Raman setup was also employed to acquire the micro-photoluminescence (μ-PL) spectra changing the grating from 1800 L/mm to 600 L/mm. In all the configurations, the laser power was filtered with a neutral density (ND) filter at 1%. 

Calculations of the MoS_2_/GaN(0001) interface were performed within the density functional theory (DFT). We used the plane-wave Quantum Espresso code [60] with Hamada’s van der Waals exchange-correlation functional [61] and standard solid-state pseudopotentials [62,63]. The latter was based on the Perdew–Burke–Ernzerhof functional [64]. To have a broader comparison with the experiment, monolayer MoS_2_ interfaces with ideal Ga-terminated GaN surfaces and with oxidized GaN surfaces were considered. A slab model comprising 16 bilayers of Ga-N, whose bottom termination was passivated with hydrogen, was used to model the GaN substrate. On the top termination instead, the Ga-terminated GaN(0001) surface interacted with the MoS_2_ layer. The quasi-commensurate lattice constants of MoS_2_ with respect to the surface vectors of GaN(0001) allowed for the construction of an interface model with unit-cell periodicity. This (1 × 1) interface model resulted in a small tensile strain for the MoS_2_ layer (1.7%), whereas the GaN substrate was unstrained. The plane-wave cut-off kinetic energy was set to 50 Ry and the augmented charge density cut-off was set to 400 Ry, respectively. A (12 × 12 × 1) Monkhorst-Pack grid [65] was used for the sampling of the Brillouin zone. To avoid spurious interactions between the periodic replicas of the system perpendicular to the interface, a vacuum space of 20 Å was inserted in the simulation supercell.

## 3. Results

**Figure 1:**
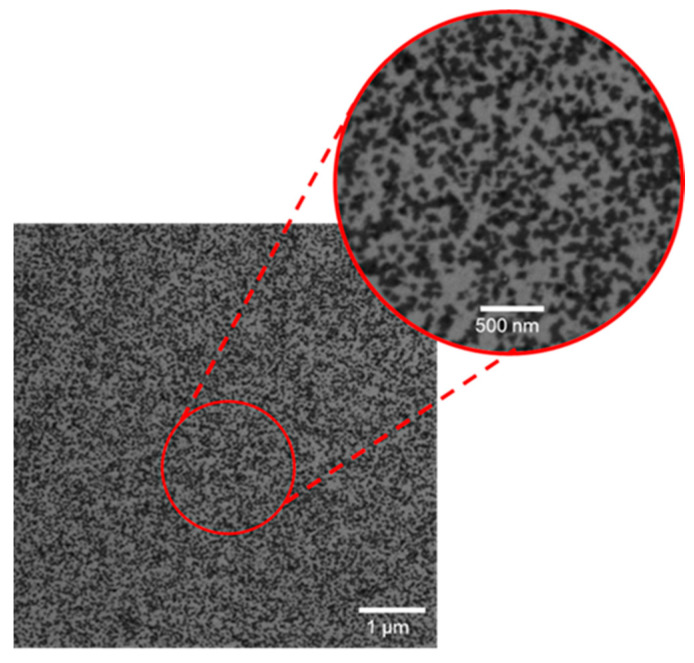
Overview SEM image and magnification (insert) of CVD-grown MoS_2_ domains on GaN.

The nucleation and growth of MoS_2_ on the GaN surface was preliminarily investigated by SEM images collected immediately after the deposition of the samples on different areas. Figure 1 shows a representative image on a 6 μm × 6 μm area, which demonstrates a very dense coverage with MoS_2_ domains (dark contrast). The insert of Figure 1 allows us to better distinguish the domain’s size and coverage, with typical sizes ranging from 50 to 150 nm and an estimated surface coverage ~35%. Furthermore, the typical triangular shape of these domains can be deduced clearly by a plan view TEM image, as reported in Figure S2 of the Supplementary Materials.

**Figure 2:**
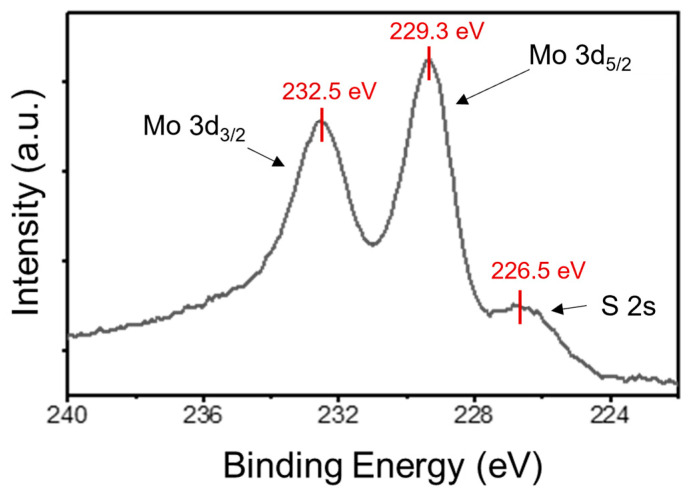
XPS spectra of Mo 3d and S 2s core levels were collected on the as-grown MoS_2_ on GaN. The binding energies of the S 2s peak (226.5 eV), Mo3d_3/2_ (232.5 eV), and Mo3d_5/2_ (229.3 eV) peaks associated with Mo atoms with Mo^4+^ oxidation are indicated.

XPS analyses provided surface-sensitive chemical information on the stoichiometry of MoS_2_ domains. A near-stoichiometric [S]/[Mo] ≈ 2 ratio was deduced by a preliminary elemental analysis. To obtain more detailed information on the Mo oxidation state and Mo-S bonding, the Mo 3d5/2, Mo 3d3/2, and S 2s core-level spectra (located at 229.3, 232.5, and 226.5 eV, respectively) were collected, as reported in Figure 2. In particular, the Mo 3d spectrum confirms that Mo atoms exhibit only the Mo4+ oxidation state associated with the 2H-MoS_2_ [66], without any contribution at higher oxidation states correlated with the presence of MoO_3_ [67]. 

**Figure 3:**
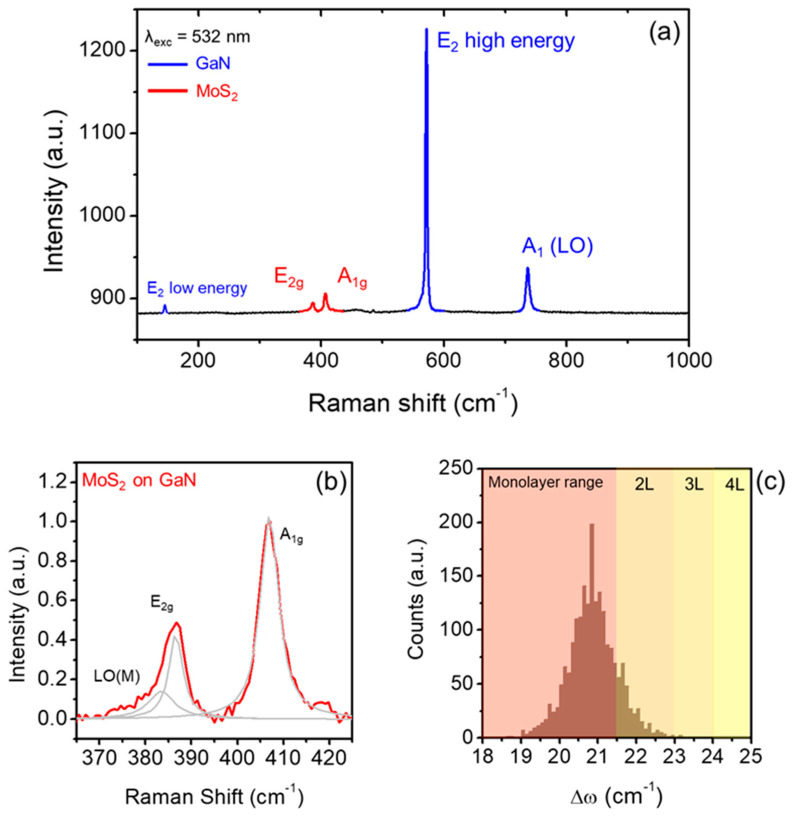
(**a**) Raman spectrum of the 2D/3D van der Waals heterostructures, showing the GaN and MoS_2_ vibrational modes by blue and red lines, respectively. (**b**) Detail on the MoS_2_ peaks after a deconvolution analysis, which pointed out the presence of a further LO(M) peak at lower wavenumbers. (**c**) Histogram of the difference between the two peaks (Δω = ω_A1g_ − ω_E2g_).

Subsequently, Raman spectroscopy was exploited to evaluate the vibrational features of the MoS_2_/GaN heterostructure. In particular, the characteristic Raman peaks of GaN, i.e., E_2_ low-high energy and A_1_(LO), and of MoS_2_, i.e., E_2g_ and A_1g_, can be observed in the wavenumber range between 100 and 1000 cm^−1^, as shown by the blue and red lines in Figure 3a. The very narrow and intense A_1_(LO) peak is consistent with the low *n*-type doping (~10^16^ cm^−3^) of the GaN substrate [36]. Focusing on the wavenumber region between 365 and 425 cm^−1^ reported in Figure 3b, a baseline subtraction and a normalization of the A_1g_ peak were applied with the purpose of extrapolating detailed information on the crystal quality of the CVD-grown MoS_2_ flakes. Despite a low-medium A_1g_/E_2g_ intensity ratio (~0.5), the two main Raman modes could be fitted by narrow single Gaussian peaks. In addition, the deconvolution analysis revealed the presence of a small LO(M) component near the E_2g_ mode, associated with defects or with the domain boundaries [68]. 

To obtain statistically relevant information, a wide number of Raman spectra were collected in a 10 μm × 10 μm sample. From this array of spectra, we evaluated the homogeneity of the MoS_2_ number of layers, by extracting the wavenumber difference of the A_1g_ and E_2g_ Raman modes (Δω = ω_A1g_ − ω_E2g_), which is known to be dependent on MoS_2_ thickness [69]. In particular, the statistical distribution of the MoS_2_ thickness was obtained from the Δω histogram in Figure 3c, which shows a mean value of Δω = 20.9 cm^−1^ with a standard deviation of 0.9 cm^−1^. This distribution shows that the MoS_2_ mainly consisted of monolayers, with a small percentage of bilayers. 

**Figure 4:**
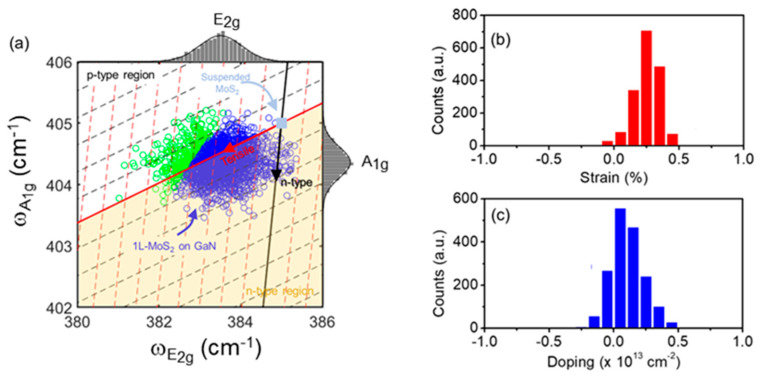
(**a**) Correlative E_2g_ vs. A_1g_ plot to evaluate the strain and doping effects induced on the MoS_2_ flakes by the growth conditions and the interaction with GaN substrate. Blue and green points in panel (**a**) correspond to the peak positions obtained in the 1L and multilayers regions, respectively. Evaluated strain (**b**,**c**) doping distributions for 1L of MoS_2_.

In addition to the thickness assessment, the A_1g_ and E_2g_ Raman peak positions provide information on the strain and doping of the thin MoS_2_ domains, according to the procedure discussed in several recent papers [9,35,70,71]. These doping and strain effects can be due to the CVD growth conditions and to the interaction with the GaN substrate. Figure 4a shows a correlative E_2g_ vs. A_1g_ plot, where the graph is separated in four quadrants by the intersection of the ideal strain (red) and doping (black) lines. The intersection point represented by the light blue square corresponds to the ideal (E_2g_, A_1g_) Raman modes of unstrained and undoped monolayer MoS_2_. To this aim, the literature values of the (E_2g_,A_1g_) peak positions ($\omega_{E2g}=385\;{cm}^{-1}\;$; $\omega_{A1g}=405$ ${cm}^{-1}$) for suspended MoS_2_ flakes [5,72] were taken as the best approximation to this ideal condition, since the effects of the interaction with substrate are excluded in that case. 

The red and black arrows indicate the directions of tensile strain and *n*-type doping regions, respectively, while the opposite side of the red and black lines correspond the compressive strain and *p*-type doping. The *n*-type doping region was indicated by the yellow area to better distinguish it from the *p*-type region (white) in the upper-side of the graph. The experimental values of the peak positions from the same array of Raman spectra used in Figure 3 are reported by the empty circles in the graph of Figure 4a. The corresponding histograms of the E_2g_ and A_1g_ peak values are also reported on the upper-side and right-side of the graph (grey bars). In Figure 4a, the blue and green points correspond to the peaks’ positions obtained in the 1L and 2L (or multilayer) regions, respectively, as determined in the histogram of Figure 3c. For 1L of MoS_2_, an average tensile strain of around 0.2% and light *n*-type doping (<0.1 × 10^13^ cm^−2^) is deduced from the correlative plot in Figure 4a. A more precise evaluation was obtained by evaluating the strain and doping for each data point of 1L-MoS_2_ and by building the histograms of the strain and doping distribution, as reported in Figure 4b,c. A tensile strain of 0.25 ± 0.10% and a *n*-type doping of (0.11 ± 0.12) × 10^13^ cm^−2^ were deduced from the mean value and the standard deviation of these two distributions. Notably, nearly unstrained monolayer MoS_2_ on GaN has been recently reported also under different CVD growth conditions, resulting in the formation of micrometer size triangular domains [42] or continuous monolayer MoS_2_ films [36,42]. These observations confirm the key role played by the low mismatch of the in-plane lattice constants (<1%) and of thermal expansion coefficients between MoS_2_ and GaN. Furthermore, the low *n*-type doping is consistent with the typically reported unintentional *n*-type doping of MoS_2_ obtained by exfoliation from bulk crystals, probably induced by the presence of native defects (such as sulfur vacancies) [73,74]. On the other hand, *n-* or *p*-type doping behavior has been reported for MoS_2_ grown by CVD approaches, depending on several factors, such as the content of MoO_3_ residues in the films (typically responsible for *p*-type doping) or the peculiar interaction with the underlying substrate. In this regard, the average *n*-type doping of the CVD-grown MoS_2_ on GaN in the present work is consistent with the absence of MoO_3_ residues, as indicated by XPS analyses in Figure 2.

**Figure 5:**
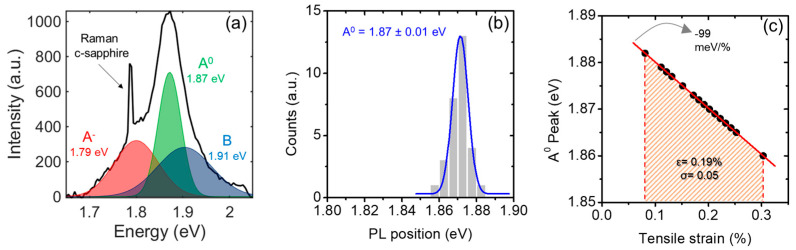
(**a**) Photoluminescence spectrum of MoS_2_ on GaN, where the two excitons (A^0^ and B) and trion (A^−^) components were extracted after deconvolution analysis. (**b**) Distribution of the A^0^ exciton peak positions evaluated at different points of the sample. (**c**) A^0^ peak positions as a function of the tensile strain.

Figure 5a shows a representative PL spectrum of the MoS_2_/GaN obtained with a laser excitation wavelength of 532 nm. The intense PL emission is a further confirmation of the good MoS_2_ crystal quality achieved by CVD. In fact, a high density of defects in MoS_2_ films would involve non-radiative recombination of excitons, causing PL quenching [75,76]. 

In detail, a deconvolution analysis performed on the spectrum of Figure 5a revealed the coexistence of three components. The A^0^ and B peaks located at 1.87 eV and 1.91 eV correspond to the excitonic emissions due to the spin-orbit coupling splitting of the MoS_2_ valence band [77]. Differently, the red-area convoluted peak at lower energy (1.79 eV) is related to the trion (also known as charged exciton) contribution, consisting of the bound state between an electron (or hole) and an exciton [78,79]. This deconvolution analysis confirms the absence of the defect-related peak X_D_, typically located at lower energy with respect to the trionic component. After a statistical analysis of different MoS_2_/GaN areas, we built a histogram of the excitonic peak energy A^0^, as reported in Figure 5b. This distribution showed a standard deviation of 10 meV around a mean value of 1.87 eV, indicating a spatially uniform PL emission from the sample surface. The energy of the main PL peak (A^0^) has been shown in the literature to be dependent on the strain of MoS_2_, with a red shift of the peak at increasing strain with a rate of −99 meV/% [72]. By applying this linear relation, the values of the tensile strain were calculated from the experimental values of the A^0^ peak energy, as reported in Figure 5c. From this analysis, strain values in the range between 0.08 and 0.3% were deduced, with a mean value of ε = 0.19 ± 0.05%, in good agreement with the previous estimation by Raman measurements.

**Figure 6:**
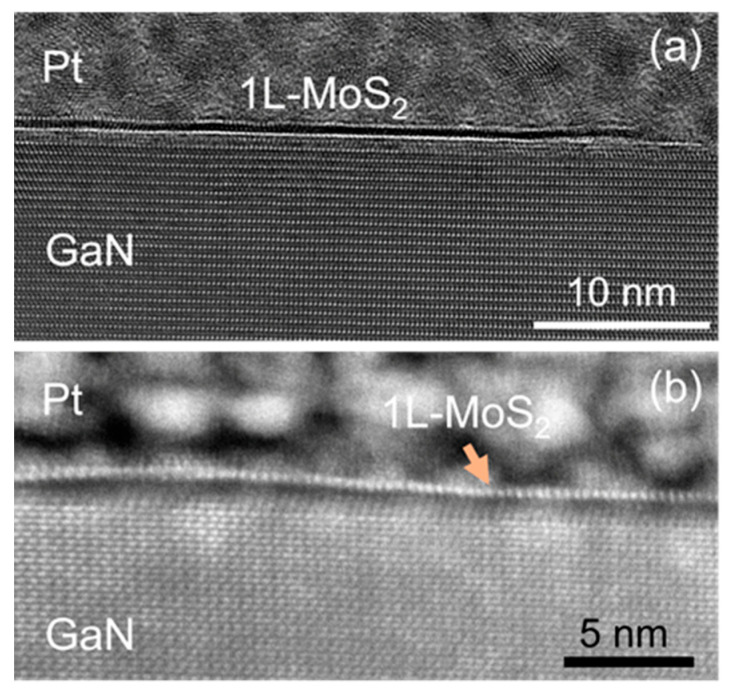
(**a**) Overview the HR-TEM image showing 1L of MoS_2_ conformal to the (0001) basal plane of GaN. (**b**) Atomic resolution STEM image showing the presence of a van der Waals gap between 1L of MoS_2_ and the underlying GaN surface.

The interface properties of the 2D/3D vdW heterostructure were characterized by cross-sectional transmission electron microscopy analyses. Figure 6a is an overview HR-TEM image, showing a monolayer MoS_2_ conformal to the crystalline GaN substrate, similarly to other reports for MoS_2_ grown by CVD approaches on GaN or other crystalline hexagonal substrates [36,37,80,81]. Furthermore, the presence of a vdW gap between the single layer of MoS_2_ and GaN surface is clearly demonstrated by the high-resolution HAADF-STEM image in Figure 6b. This is a direct indication of a weak bond between MoS_2_ and the underlying GaN crystal. Notably, this high-resolution STEM analysis reveals a different structure of the first crystalline planes of GaN with respect to the underlying bulk crystal. As reported in previous structural investigations of CVD MoS_2_/GaN heterostructures [37], such differences can be attributed to partial oxidation of the GaN surface during the MoS_2_ growth process or some form of surface reconstruction.

In the last section of this paper, DFT calculations have been performed to obtain a deeper physical understanding of the type of interaction and electronic properties of the MoS_2_/GaN interface. In this context, it is worth mentioning that DFT calculations of this kind of heterostructure have been recently reported in the literature [57,59]. A S-Ga equilibrium distance of 0.232 nm in Ref. [57] and 0.237 nm in Ref. [59] was evaluated for the ideal case of a lattice-matched interface between MoS_2_ and Ga-terminated GaN, indicating the formation of a covalent-like bond at the interface. Clearly, those calculation results do not match with the results of atomic resolution TEM analyses of the MoS_2_/GaN heterostructure obtained in the present work and with those recently reported by different research groups [37,47], which showed the presence of a larger vdW gap separating S from Ga atoms. 

As a matter of fact, under real experimental conditions, the GaN(0001) surface can be subjected to reconstructions or to oxidations. Hence, to provide a more complete description of the MoS_2_/GaN system, we performed DFT calculations of the heterostructure considering three different model configurations of the GaN surface (see Figure S3 of the Supplementary Materials): (i) the ideal Ga-terminated GaN, analogous to the one reported in the literature; (ii) the passivation of the Ga termination with an oxygen coverage of one monolayer; and (iii) the formation of an ultra-thin crystalline Ga_2_O_3_ oxide. The analysis of the DFT predictions for these three configurations has been compared with the experimental results for our system.

**Figure 7:**
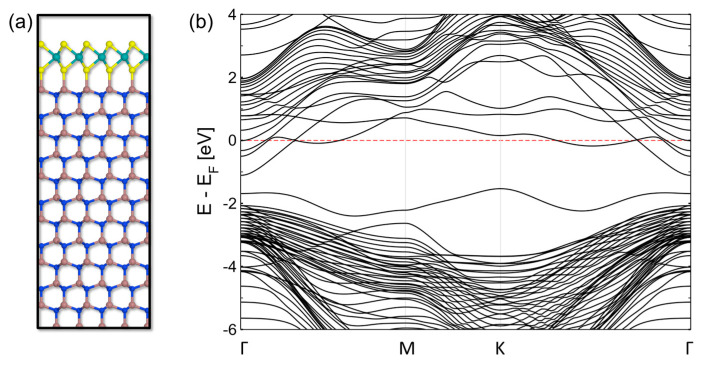
(**a**) Structure of the interface between Ga-terminated GaN and monolayer MoS_2_ seen from the ($11\bar{2}0$) plane and (**b**) the energy band structure of this heterostructure. E_F_ refers to the calculated Fermi level of the system.

Figure 7a shows the most stable configuration obtained by DFT calculations of the ideal Ga-terminated GaN surface, where a Ga-S equilibrium distance of 0.241 nm was estimated, in close agreement with recent literature reports [57,59]. Furthermore, the calculated band structure for this system (reported in Figure 7b) shows a high *n*-type doping of MoS_2_ and strong perturbation of its energy bands. As a matter of fact, this ideal configuration of the MoS_2_/GaN(0001) interface does not provide a real representation of the system. For this reason, we performed DFT calculations considering the presence of O atoms bonded to the GaN(0001) surface.

**Figure 8:**
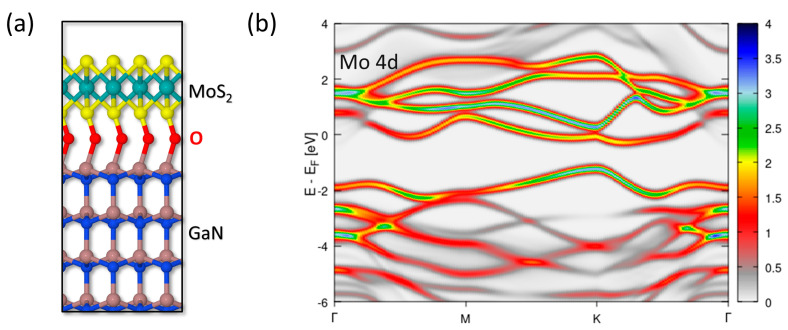
(**a**) Structure of the MoS_2_/GaN interface with oxygen passivation of Ga terminations with monolayer O surface coverage, seen from the ($1\bar{1}00$) plane. (**b**) Electronic contributions of Mo *d* orbitals in the wavevector-resolved projected density of states along the Γ-M-K-Γ Brillouin zone path. E_F_ refers to the calculated Fermi level of the system.

Figure 8a,b shows the results for oxygen-passivated Ga terminations with one monolayer O surface coverage. Also in this case, a covalent interface interaction was obtained, which again deviates from the experimental evidence of a weak van der Waals bonding. The theoretically calculated strong interface coupling had a structural impact only on the topmost Ga layer of the substrate and perturbed the MoS_2_ bands with respect to those of a freestanding MoS_2_ layer (see Figure S4 of the Supplementary Materials). 

**Figure 9:**
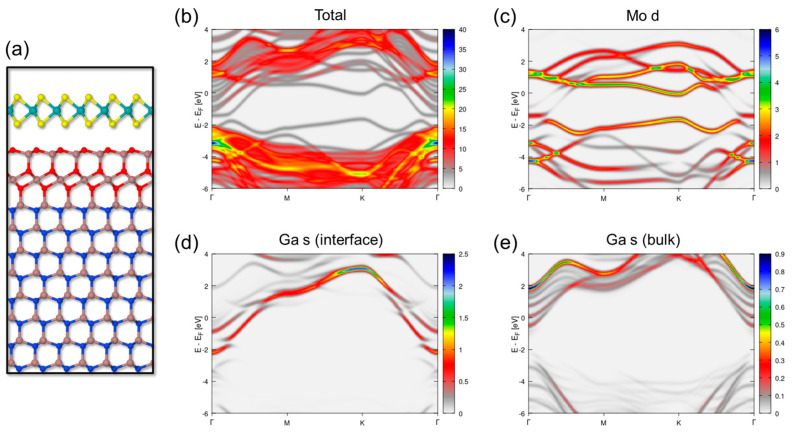
(**a**) Structure of the oxidized MoS_2_/GaN(0001) interface, showing the formation of an ultra-thin layer of native oxide Ga_2_O_3_, seen from the ($11\bar{2}0$) plane. Wavevector-resolved projected density of states along the Γ-M-K-Γ Brillouin zone path for (**b**) all electronic states, (**c**) contributions of Mo d orbitals, (**d**) contributions of surface Ga s orbitals, and (**e**) contributions of bulk Ga s orbitals. E_F_ refers to the calculated Fermi level of the system.

We thereon considered the formation of an ultra-thin layer of surface native oxide Ga_2_O_3_, which significantly reduces the surface energy of GaN(0001) as compared to other oxidized reconstructions [82]. This layer is characterized by an O−Ga−O trilayer which inverts the polarization of the GaN layer along the [0001] direction, followed by a Ga−O bilayer that terminates the oxidized surface (Figure 9a). The interaction of this Ga_2_O_3_-terminated GaN surface with MoS_2_ gave rise to a van der Waals interface with an oxygen-sulfur interface distance of 3.05 Å. This distance is significantly larger than the one reported in the literature for the ideal MoS_2_/GaN(0001) system [57,59] and it is in better agreement with the experimental observations of a vdW gap at the interface. Figure 9b–e shows the total and partial electronic contributions (Mo d and Ga s orbitals) in the density of states of the heterosystem, plotted at the close Γ-M-K-Γ path of the Brillouin zone. The pristine bands of MoS_2_ are clearly preserved in this case, showing a direct band gap of 1.7 eV at the K point of the Brillouin zone. We note that such an interface induces a significant *n*-type doping for the MoS_2_ sheet, due to a shift of surface Ga s states deriving from the oxide layer towards lower energies (because of Ga-O bonding). Such a shift brings the Fermi level of the system close to the conduction band of the MoS_2_ layer. Overall, the theoretical calculations indicate that a van der Waals interface at the MoS_2_/GaN(0001) heterosystem is expected when an ultra-thin Ga_2_O_3_ native oxide forms at the substrate’s surface, whereas it is rather improbable for low surface oxygen coverages.

## 4. Conclusions

The structural, chemical, vibrational, and light emission properties of the CVD-grown MoS_2_ heterostructure with GaN have been investigated in detail by several microscopic and spectroscopic techniques and by DFT calculations. XPS analyses on as-grown samples showed the formation of stoichiometric MoS_2_. According to micro-Raman spectroscopy, monolayer MoS_2_ domains on GaN exhibit an average *n*-type doping of (0.11 ± 0.12) × 10^13^ cm^−2^ and a small tensile strain ε ≈ 0.25%), whereas an intense light emission at 1.87 eV was revealed by PL analyses. Furthermore, a gap at the interface was shown by cross-sectional TEM analysis, confirming the vdW bond between MoS_2_ and GaN. Finally, DFT calculations of the heterostructure were carried out, considering three different configurations of the interface, i.e., (i) an ideal Ga-terminated GaN(0001) surface, (ii) the passivation of the Ga surface by a monolayer of oxygen, and (iii) the presence of an ultrathin Ga_2_O_3_ layer. This latter configuration is the only one which accounts for the formation of a vdW bond at the interface and a significant *n*-type doping of MoS_2_, in agreement with the experimental observations. These results provide an insight on the MoS_2_/GaN interfacial properties, which rule the current injection mechanisms across these vdW heterostructures. Further studies on how to tailor the structural/chemical properties of this interface will be crucial for future applications in electronics and optoelectronics.

## Data Availability

The data are available on reasonable request from the corresponding author.

## Supplementary Materials

The following supporting information can be downloaded at https://www.mdpi.com/article/10.3390/nano14020133/s1. Figure S1: AFM image of GaN-on-c-sapphire with an RMS of about 0.3 nm. Figure S2: In-plane TEM image of MoS2 triangular flakes on a carbon grid. Figure S3: Interface models used for the DFT calculations of the MoS2/GaN heterostructure, considering three different configurations of the GaN surface. Figure S4: Band structure of freestanding MoS_2_ based on DFT calculations.
