# Supplementary material for: Interface Properties of MoS2 van der Waals Heterojunctions with GaN
Source: Nanomaterials (Basel). 2024 Jan 5;14(2):133. doi: 10.3390/nano14020133 (PMC10818867; doi:10.3390/nano14020133)
Supplement: Supplementary file 1 [file nanomaterials-14-00133-s001.zip › nanomaterials-2771108-supplementary.pdf]

Supporting Information

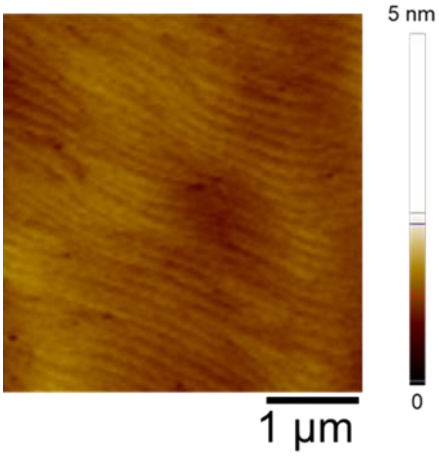

Figure S1: AFM image of GaN-on-c-sapphire with a RMS of about 0.3 nm.

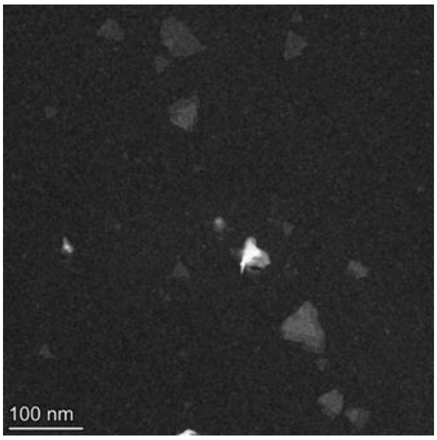

Figure S2: In-plane TEM image of MoS_2 triangular flakes on a carbon grid.

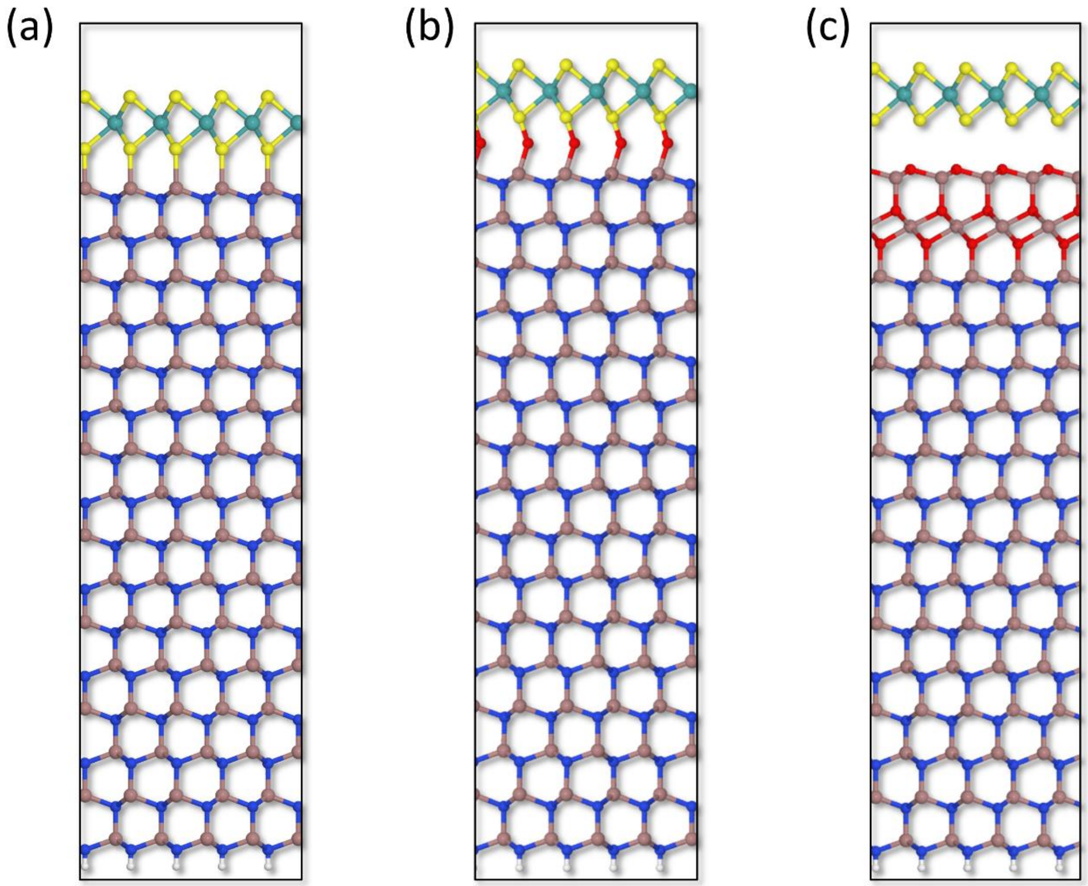

Figure S3: Interface models used for the DFT calculations of the MoS_2/GaN heterostructure, considering three different configurations of the GaN surface: (**a**) ideal Ga-terminated GaN; (**b**) passivation of the Ga termination with one monolayer coverage of O atoms; (**c**) the formation of an ultra-thin crystalline Ga_2O_3 oxide.

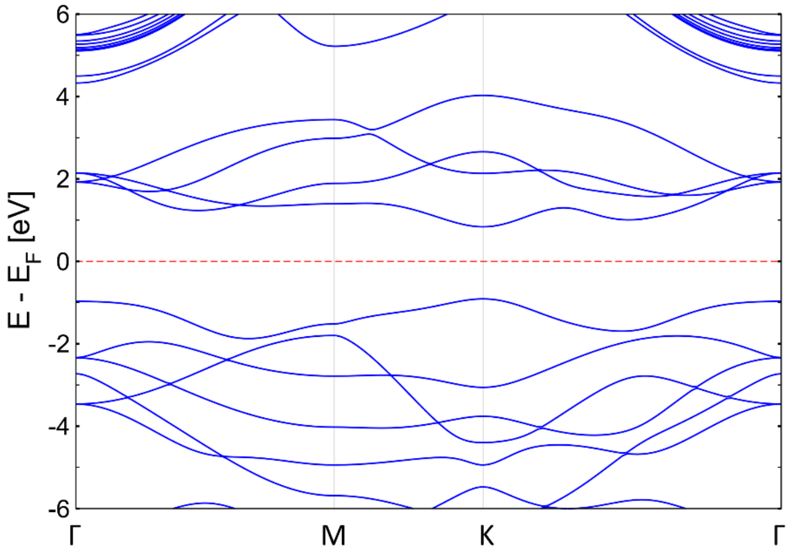

Figure S4: Band structure of freestanding monolayer MoS_2 based on DFT calculations.
